# Characteristics of leptospirosis with systemic inflammatory response syndrome: a multicenter study

**DOI:** 10.1186/s12941-015-0117-x

**Published:** 2015-12-21

**Authors:** Hava Yilmaz, Vedat Turhan, Kadriye Kart Yasar, Mustafa Hatipoglu, Mustafa Sunbul, Hakan Leblebicioglu

**Affiliations:** Department of Infectious Diseases and Clinical Microbiology, School of Medicine, Ondokuz Mayis University, 55139 Samsun, Turkey; Department of Infectious Diseases and Clinical Microbiology, Haydarpasa Training Hospital, Gulhane Military Medical Academy, Istanbul, Turkey; Department of Infectious Diseases and Clinical Microbiology, Bakırköy Sadi Konuk Training and Research Hospital, Istanbul, Turkey

**Keywords:** Leptospirosis, SIRS, Prognosis

## Abstract

**Background:**

Leptospirosis is a common zoonotic infection in the world. In patients with leptospirosis, in case of presence of Systemic Inflammatory Response Syndrome (SIRS), clinical and laboratory findings can be mistaken for sepsis due to other causes of infection. The purpose of this study is to assess the clinical and laboratory parameters of patients with leptospirosis in terms of the presence of SIRS and to examine the association of these with mortality.

**Methods:**

One hundred fifty-seven patients were included in the study. The patients were classified according to the presence or absence of SIRS and divided into SIRS (+) and SIRS (−). Patient files were retrospectively evaluated. Clinical features and laboratory data were compared, and risk factors associated with mortality were determined.

**Results:**

SIRS (+) was found in 70 % (*n* = 110) of patients who had signs on admission. Comparison of the clinical symptoms and findings of organ systems in the SIRS (+) and SIRS (−) showed that abdominal pain and vomiting were significantly more common in the SIRS (+) than in the SIRS (−) (*p* = 0.025 and *p* = 0.046, respectively). BUN and serum creatinine levels were significantly higher in the SIRS (+) than in the SIRS (−) (*p* = 0.002 and *p* < 0.001, respectively). In follow-up posterior-anterior chest radiography, pathological findings improved in 58.8 % (*n* = 40) of patients in the SIRS (+) and 27.3 % (*n* = 9) of patients in the SIRS (−) (*p* = 0.003). The mortality rate of the SIRS (+) and SIRS (−) was not significantly different (*p* = 0.868).

**Conclusion:**

In patients with positive SIRS findings, while examining the etiology of sepsis, leptospirosis should come to mind especially in endemic areas for differential diagnosis. Early initiation of antibiotic and supportive therapy can be lifesaving in these patients.

## Background

Leptospirosis is a systemic bacterial infection caused by the Leptospira genus. The most frequent modes of transmission of the infection to humans are consumption of food and water contaminated with saliva, urine, and feces of infected animals and penetration of mucous membranes or broken skin [1]. The most consistent pathological finding in leptospirosis is vasculitis [2]. Leptospira can escape the immune response of the host and survive in various tissues, giving rise to systemic disease in some cases. Leptospira was shown to cause septicemia and vessel injury by an unexplained mechanism [3]. Histopathological changes, including generalized vasculitis affecting the kidney, lung, liver, brain, and meninges, were observed in microscopic examinations [4]. Coagulation disorders resulting in hemorrhages and multi-organ failure can occur in patients with leptospirosis and sepsis [5]. Systemic Inflammatory Response Syndrome (SIRS) is a systemic response to infection trauma burns or other conditions such as cancer with symptoms including fever tachycardia, tachypnea and leukocytosis. Sepsis is defined as presence of (probable or documented) infection together with systemic manifestations of infection [6].

Thus, the purpose of our study is to assess the clinical and laboratory parameters of patients with leptospirosis in terms of the presence of SIRS and to examine the association of these with mortality.

## Methods

Data on patients admitted to the Department of Clinical Microbiology and Infectious Diseases of Ondokuz Mayis University Hospital, Gulhane Military Medical Academy, Haydarpaşa Training Hospital, and Bakirkoy Sadi Konuk Training and Research Hospital between January 1991 and January 2013 were included in the study. This study was approved by Ondokuz Mayis University Ethics Committee.

### Data collection and laboratory analyses

Demographic data (age, sex, profession), epidemiological data (contact with rodent, place of residence), and symptoms and findings related to the disease (fever, vomiting, diarrhea, headache, stomach ache, myalgia, icterus, oliguria, respiratory and heart rate, cough, mental changes, neck stiffness, hemorrhage, redness of conjunctiva, hepatomegaly, hemodialysis, radiologic lung findings) were recorded. The duration between the onset of symptoms and admission to the hospital, treatment and hospitalization duration, and use of medications were recorded, and the prognoses of the patients were evaluated.

The laboratory investigation included a complete blood count and assessment of coagulation parameters (prothrombin time [PT] and activated partial thromboplastin time [aPTT]). Serum biochemical measurements included leukocyte and platelet counts, hemoglobin, serum potassium, aspartate aminotransferase (AST), alanine aminotransferase (ALT), creatinine, blood urea nitrogen (BUN), creatinine phosphokinase, total and direct bilirubin and alkaline phosphatase (ALP), C-reactive protein (CRP), and erythrocyte sedimentation rate (ESR) levels. Chest radiography was performed to determine the presence and type of infiltration.

The sera of the patients were tested for leptospira antibodies with an enzyme-linked immunosorbent assay (ELISA) (Virion ELISA, Institut Virion GmbH, Würzburg, Germany and PanBio ELISA, Brisbane, Australia) [7]. Blood samples taken from the patients were cultured in Leptospira Ellinghausen-McCullough-Johnson-Harris medium (Difco) for generation of leptospira species.

### Definition of leptospirosis

The diagnosis of leptospirosis was made according to criteria published by the Centers for Diseases Control and Prevention (CDC) in 2013 [8]. Patients with supportive and confirmed laboratory criteria according to the CDC guidelines for leptospirosis were included in the study. Those without a supportive and/or confirmed laboratory diagnosis were excluded from the study [8]. Microscopic agglutination test (MAT) was performed in the spirochete laboratory of Etlik Veterinary Central Control and Research Institute (ECVCRI), Ankara, Turkey. Macrotube agglutination (Danke-Seien, Japan) tests were performed in the Clinical Microbiology Laboratory of Cerrahpasa Medical Faculty of Istanbul University.

#### Supportive criteria

A *Leptospira* agglutination titer of ≥200 but <800 in the MAT in one or more serum specimens, or demonstration of anti-*Leptospira* antibodies in a clinical specimen by indirect immunofluorescence, or demonstration of *Leptospira* in a clinical specimen by dark-field microscopy, or detection of Immunoglobulin (Ig) M antibodies against *Leptospira* in an acute phase serum specimen.

#### Confirmed criteria

Isolation of *Leptospira* from a clinical specimen, *or* a four-fold or greater increase in *Leptospira* agglutination titers between acute- and convalescent-phase serum specimens studied at the same laboratory, *or* demonstration of *Leptospira* in tissue by direct immunofluorescence, *or* a *Leptospira* agglutination titer of ≥800 in the MAT in one or more serum specimens, *or* detection of pathogenic *Leptospira* DNA (e.g., by PCR) in a clinical specimen [8]. All the patients tested positive for at least one of according to the CDC’s criteria for leptospirosis.

### Definitions of SIRS and sepsis

The diagnosis of SIRS was made according to the criteria of the Surviving Sepsis Campaign: (a) body temperature >38 °C or <36 °C, (b) heart rate >90 beats/min, (c) respiratory rate >20 breaths/min or PaCO_2_ < 32 mmHg, (d) white blood cell count >12,000 cells/mm^3^ or <4000 cells/mm^3^, or >10 % immature white blood cells. The presence of SIRS accompanied by infection was diagnosed as sepsis [6]. Based on the presence of findings associated with SIRS, all the patients were divided into two main groups: SIRS (+) and SIRS (−).

### Statistical analysis

IBM SPSS for Windows (version 21.0) was used for data management and statistical analysis. Descriptive values are expressed as mean ± SD for variables with a normal distribution or medians and interquartile ranges for variables with an abnormal distribution. Discrete variables were expressed as numbers and percentages. The Kolmogorov–Smirnov test was used to analyze the normal distribution of the variables. Following normality testing, independent *t* tests and a Mann–Whitney *U* test were used to compare continuous data. The Chi-square test was used in the analysis of categorical data. Multivariate logistic regression analysis was used to find independent covariates. A *p* value less than 0.05 was regarded as significant.

## Results

### Demographic data of patients

The study consisted of 157 patients, with 129 (82 %) males and 28 (17 %) females. The mean age was 42 ± 18 (range 18–75). One hundred (63 %) patients were from the Black Sea region, and 57 (37 %) were from different cities in the Marmara region. The occupational distribution was as follows: 63 (40.1 %) were farmers, 25 (16 %) were workers [10 (40 %) were hod carrier, 7 (28 %) were sewage workers, 5 (20 %) were forest workers, 3 (12 %) were textile workers], 22 (14 %) were military personnel, and 18 (11.5 %) were housewives. Ten (6.3 %) patients were porters, and 19 (12.1 %) were classified as “other occupations”. Seventy-two (45.8 %) of the patients had a history of contact with rodents.

### Diagnosis of leptospirosis

Analysis of the patients according to the CDC criteria for leptospirosis revealed definitive laboratory findings in 31 (19.7 %) patients and supportive laboratory findings in 126 (80.3 %) patients. The spiral form of leptospira was detected with dark field microscopy in the blood of 116 (74.4 %) patients. Ig M positivity was detected in 128 (82.1 %) patients. Thirty-one (19.7 %) patients had a titer >200 in the MAT test, and 52 (33.1 %) had a titer <200. Only 9 (11.1 %) patients with sera positive findings by MAT had a titer ≥800. In 37 (25.5 %) patients diagnosed according to MAT. Culture was formed from the blood samples of 34 patients. Leptospira bacteria were isolated in 22 (14 %) of these cultures. In all patients whose MAT was positive, common serological leptospira subtypes were as follows: *L. biflexa* serovar Patoc (42.1 %, *n* = 35), *L. interrogans* serovar icterohaemorragia (31.3 %, *n* = 26), *L. interrogans* serovar bratislava (12 %, *n* = 10), and *L. interrogans* serovar grippotyhos (6 %, *n* = 5). In the macrotube agglutination analysis, the rates of *L. interrogans* serovar icterohaemorragia and *L. interrogans* serovar autumnalis were 64.9 % (*n* = 24) and 35.1 % (*n* = 13), respectively.

### Evaluation of patients according to SIRS

SIRS (+) was found in 70 % (*n* = 110) of patients who had signs on admission. Comparison of the clinical symptoms and findings of organ systems in the SIRS (+) and SIRS (−) showed that abdominal pain and vomiting were significantly more common in the SIRS (+) than in the SIRS (−) (*p* = 0.025 and *p* = 0.046, respectively). Additional clinical symptoms and findings are summarized in Table 1. BUN and serum creatinine levels were significantly higher in the SIRS (+) than in the SIRS (−) (*p* = 0.002 and *p* < 0.001, respectively). The need for hemodialysis was not different between the SIRS (+) and SIRS (−) (*p* = 0.128). In follow-up posterior-anterior chest radiography, pathological findings improved in 58.8 % (*n* = 40) of patients in the SIRS (+) and 27.3 % (*n* = 9) of patients in the SIRS (−) (*p* = 0.003). Additional laboratory symptoms and findings are summarized in Table 2.

**Table 1:** Detailed clinical symptoms of the SIRS (+) and SIRS (−)

Features	SIRS (+) (n = 110) *n* (%)	SIRS (−) (n = 47) *n* (%)	P values
Myalgia	71 (65.1)	29 (61.7)	0.682
Jaundice	65 (59.1)	34 (72.3)	0.115
Hemorrhage	24 (21.8)	7 (14.9)	0.386
Conjunctival petechiae	28 (25.5)	9 (19.1)	0.538
Headache	55 (50.5)	23 (48.9)	0.861
Vomiting	74 (67.9)	24 (51.1)	*0.046*
Cough	37 (33.9)	11 (23.4)	0.257
Abdominal pain	54 (49.1)	14 (29.8)	*0.025*
Diarrhea	33 (30.3)	11 (23.4)	0.441
Oliguria	44 (40.4)	14 (30.4)	0.279
Change in mental status	28 (25.5)	10 (22.2)	0.837
Radiological lung findings	40 (58.8)	9 (27.3)	*0.003*
Neck stiffness	8 (18.6)	4 (13.8)	0.751
Hemodialysis	18 (16.4)	3 (6.7)	0.128
Hepatomegaly	38 (34.5)	20 (42.6)	0.370
Mortality	13 (11.8)	6 (12.8)	0.868

Italic values indicate statistically significant (p < 0.05)

**Table 2:** Laboratory findings of the SIRS (+) and SIRS (−) patients [median (IQR), *n* (%), mean ± SD]

Features	SIRS (+) (n = 110)	SIRS (−) (n = 47)	P values
Hemoglobin (g/dl)	11.55 ± 2.56	12.33 ± 2.11	0.097
Leukocyte (/mm^3^)	12,700 (1400–44,100)	8900 (1800–33,100)	<*0.001*
Platelet (/mm^3^)	90,000 (2800–450,000)	113,000 (17,000–88,000)	0.108
Total bilirubin (mg/dL)	6.3 (0.1–83)	3.7 (0.2–54)	0.077
Direct bilirubin (mg/dL)	3.3 (0.1–50)	2.2 (0.1–37)	0.065
ALP (U/L)	247 (47–1594)	22.0 (39–1700)	0.592
BUN (mg/dL)	73 (5–474)	35 (5–153)	*0.002*
Creatinine (mg/dL)	4.1 (0.1–47)	1.4 (0.5–9.7)	<*0.001*
Potassium (mg/dL)	4 (2.1–66)	3.8 (2.6–5.2)	0.138
CPK (U/L)	312 (19–13,043)	534 (15–2667)	0.366
AST (U/L)	90.0 (15–2797)	79.0 (24–1063)	0.463
ALT (U/L)	85.5 (19–1656)	72 (17–1575)	0.441
CRP (mg/L)	27 (2–208)	38.0 (3–208)	0.195
PT, aPTT	42 (38.5)	8 (23.5)	0.149
ESR (mm/h)	52.86 ± 32.68	51.2 ± 34.83	0.798

Italic values indicate statistically significant (p < 0.05)

*IR* interquartile range, *SD* standard deviation, *ALP* alkaline phosphatase, *BUN* blood urea nitrogen, *CPK* creatinine phosphokinase, *ALT* alanine aminotransferase, *AST* aspartate aminotransferase, *CRP* C-reactive protein, *PT* prothrombin time, *aPTT* activated partial thromboplastin time, *ESR* erythrocyte sedimentation rate

The mean durations of complaints, treatment, and hospitalization were 7 ± 5, 7 ± 9, and 13 ± 11 days, respectively, in the SIRS (+) and 8 ± 6, 10 ± 5, and 12 ± 13 days, respectively, in the SIRS (−). There was no significant difference in the mean duration of the complaints, treatment, and hospitalization between the SIRS (+) and SIRS (−) (*p* > 0.05). Crystalized penicillin (33.8 %; *n* = 53), doxycycline (16.9 %; *n* = 27), ampicillin/sulbactam (21.6 %; *n* = 34), and ceftriaxone (12.8 %; *n* = 20) were commonly used to treat the patients.

### The evaluation of mortality

Nineteen out of 157 patients died (12 %). The mortality rate of the SIRS (+) and SIRS (−) was not significantly different (*p* = 0.868) (Table 1).

## Discussion

Patients with leptospirosis, in case of presence of SIRS, clinical and laboratory findings can be mistaken for sepsis caused by other infection factors [9, 10]. In 110 (70 %) of our patients, SIRS was found to be positive. SIRS findings can present early in both leptospirosis and other bacterial sepsis. Patients who are admitted to a healthcare facility and who are found to have SIRS positive findings, leptospirosis should come to mind if the patient has a history of contact with rodent or epidemiological background [4]. It is probable for some of these patients to have sepsis. However, in order to be able to diagnose SIRS positive patients with sepsis, the agent should be reproduced in the blood culture. The culture of leptospira bacteria is difficult and time consuming since it requires special media for culture and reproduces late besides the technical difficulties of conducting the procedure. It was possible to culture in only one of the three centers that participated in the study and leptospira bacteria were isolated in 22 (14 %). Thirteen (59 %) of the patients with positive culture were diagnosed with sepsis since they had SIRS (+). The high positive blood culture rates of 65 % in the centre where blood culture was conducted highlights how important it is to develop means of culture for the differential diagnosis of leptospirosis in countries where leptospira is frequent.

In the course of sepsis, presence of organ involvement is called sepsis syndrome. Leptospirosis may cause pathological changes in a great number of organs due to the endothelium damage it causes [9, 11]. However, these changes are reversible unlike the organ damage in sepsis. In our study, when SIRS (+) patients were compared with SIRS (−) patients, high urea and creatinine values at first admission, gastrointestinal system findings (stomach pain, throwing up) and pathological findings in lung radiology were found to be statistically significant (p < 0.05). Organ involvement is a finding of sepsis syndrome. Organ and system involvement, especially kidney involvement, has been found in SIRS (+) leptospirosis patients. It is difficult to find out how many of these cases are associated with sepsis.

Seguro et al. showed renal damage and pulmonary damage in the experimental leptospirosis and sepsis animal models that they conducted [12]. Acute renal failure is frequent in patients with leptospirosis. Renal failure usually develops in leptospirosis cases following acute interstitial nephritis and tubular and microvascular damage. Cengiz et al. examined 36 leptospirosis patients and reported that 65 and 51 % of cases had acute renal failure and were non-oliguric, respectively. In addition, serum BUN, creatinine, AST, ALT, bilirubin, and potassium levels were higher in oliguric patients than in non-oliguric ones (*p* < 0.005). Thirteen (48 %) patients required renal replacement therapy, and 92 % of patients recovered fully after 3–5 weeks [13].

Renal damage is one of the organ dysfunctions in sepsis [12]. Jayakumar et al. examined the reasons of acute renal failure in India, and reported sepsis as 8.8 % and leptospirosis as 7.5 % [14]. In the present study, BUN and serum creatinine levels were significantly higher in the SIRS (+) than in the SIRS (−). The need hemodialysis was greater in the SIRS (+) (*p* = 0.128). Lung injury can also occur in leptospirosis cases like sepsis. As shown in many studies, severe pulmonary injury is one of the major causes of death in Leptospirosis [15–17]. Gouveia et al. observed severe lung injury in 74 % of fatal cases of leptospirosis [18]. Pulmonary injury increases the need for mechanical ventilation and intensive care [19]. It was detected that, pulmonary involvement was significantly higher in the SIRS (+) compared to the SIRS (−) (*p* = 0.003).

Renal and pulmonary pathological findings in patients with SIRS (+) should be assessed carefully and these findings should be considered for sepsis in leptospirosis. The clinician should be careful with suitable and fast antibiotics treatment as well as the need for supplementary treatment. The management of risk factors that may affect the clinical follow-up, treatment, and prognosis of patients with leptospirosis is important. Many researchers have investigated risk factors for mortality. Some studies reported that dyspnea, oliguria, white blood cell, abnormal repolarization on an electrocardiogram, and the presence of alveolar infiltrates were mortality-related risk factors [15, 18, 19]. Esen et al. reported that a change in mental status and hyperkalemia were risk factors for mortality [20]. In our study, no statistically significant difference was found in mortality in terms of SIRS. According to these results, the presence of SIRS is not a factor that predicts mortality. However, since our study is retrospective, not having assessed comorbid situations that may affect mortality is a limitation.

As conclusion; the present study confirms that the clinical and laboratory findings of leptospirosis are similar to those of sepsis. Sepsis and leptospirosis patients should be followed closely in terms of multi-organ involvement. In areas where leptospirosis is endemic, leptospirosis should come to mind while examining the SIRS/sepsis etiology.

## References

[1] Sethi S, Sharma N, Kakkar N, Taneja J, Chatterjee SS, Banga SS (2010). Increasing trends of leptospirosis in northern India: a clinico-epidemiological study. PLoS Negl Trop Dis..

[2] Bharti AR, Nally JE, Ricaldi JN, Matthias MA, Diaz MM, Lovett MA (2003). Peru-United States Leptospirosis Consortium. Leptospirosis: a zoonotic disease of global importance. Lancet Infect Dis..

[3] Dutta TK, Christopher M (2005). Leptospirosis—an overview. J Assoc Physicians India.

[4] Lim VK (2011). Leptospirosis: a re-emerging infection. Malays J Pathol.

[5] Proulx F, Gauthier M, Nadeau D, Lacroix J, Farrell CA (1994). Timing and predictors of death in pediatric patients with multiple organ system failure. Crit Care Med.

[6] http://www.cdc.gov/nchs/data/icd/icd9cm_guidelines_2011.pdf.

[7] Pappas MG, Ballou WR, Gray MR, Takafuji ET, Miller R, Hockmeyer WT (1985). Rapid serodiagnosis of leptospirosis using the IgM-Specific Dot-ELISA; comparison with the microscopic agglutination test. Am J Trop Med Hyg.

[8] http://wwwn.cdc.gov. Accessed 5 April 2014.

[9] Dellinger R, Levy MM, Rhodes A, Annane D, Gerlach H, Opal SM (2013). Surviving Sepsis Campaign: international guidelines for management of severe sepsis and septic shock: 2012. Crit Care Med.

[10] Levett PN (2001). Leptospirosis. Clin Microbiol Rev.

[11] Hotchkiss RS, Karl IE (2003). The pathophysiology and treatment of sepsis. N Engl J Med.

[12] Seguro AC, Andrade L (2013). Pathophysiology of leptospirosis. Shock..

[13] Cengiz K, Sahan C, Sunbul M, Leblebicioglu H (2002). Acute renal failure in Leptospirosis in the black sea region in Turkey. Int Urol Nephrol.

[14] Jayakumar M, Prabahar MR, Fernando EM, Manorajan R, Venkatraman R, Balaraman V (2006). Epidemiologic trend changes in acute renal failure—a tertiary center experience from South India. Ren Fail.

[15] Doudier B, Garcia S, Quennee V, Jarno P, Brouqui P (2006). Prognostic factors associated with severe leptospirosis. Clin Microbiol Infect.

[16] Ruwanpura R, Rathnaweera A, Hettiarachchi M, Dhahanayake K, Amararatne S (2012). Severe pulmonary leptospirois associated with high fatality rate: an autopsy series in galle, southern sri lanka. Med J Malaysia.

[17] Dolhnikoff M, Mauad T, Bethlem EP, Carvalho CR (2007). Pathology and pathophysiology of pulmonary manifestations in leptospirosis. Braz J Infect Dis..

[18] Gouveia EL, Metcalfe J, de Carvalho AL, Aires TS, Villasboas-Bisneto JC, Queirroz A (2008). Leptospirosis-associated severe pulmonary hemorrhagic syndrome, Salvador, Brazil. Emerg Infect Dis..

[19] Dupont H, Dupont-Perdrizet D, Perie JL, Zehner-Hansen S, Jarrige B, Daijardin JB (1997). Leptospirosis: prognostic factors associated with mortality. Clin Infect Dis.

[20] Esen S, Sunbul M, Leblebicioglu H, Eroglu C, Turan D (2004). Impact of clinical and laboratory findings on prognosis in leptospirosis. Swiss Med Wkly..

